# Autoantibodies to Erythropoietin Receptor and Clinical Outcomes in Patients With Type 2 Diabetes and CKD: A *Post Hoc* Analysis of CREDENCE Trial

**DOI:** 10.1016/j.ekir.2023.11.024

**Published:** 2023-12-01

**Authors:** Akihiko Koshino, Brendon L. Neuen, Megumi Oshima, Tadashi Toyama, Akinori Hara, Clare Arnott, Bruce Neal, Meg Jardine, Sunil V. Badve, Kenneth W. Mahaffey, Carol Pollock, Michael K. Hansen, Takashi Wada, Hiddo J.L. Heerspink

**Affiliations:** 1 Department of Clinical Pharmacy and Pharmacology, University of Groningen, University Medical Center Groningen, Groningen, the Netherlands; 2 Department of Nephrology and Rheumatology, Graduate School of Medical Sciences, Kanazawa University, Ishikawa, Japan; 3 The George Institute for Global Health, University of New South Wales Sydney, Sydney, New South Wales, Australia; 4 Department of Cardiology, Royal Prince Alfred Hospital, Sydney, New South Wales, Australia; 5 School of Public Health, Imperial College London, UK; 6 NHMRC Clinical Trials Centre, University of Sydney, New South Wales, Australia; 7 Concord Repatriation General Hospital, Sydney, New South Wales, Australia; 8 Department of Nephrology, St George Hospital, Sydney, New South Wales, Australia; 9 University of New South Wales, Sydney, New South Wales, Australia; 10 Stanford Center for Clinical Research, Stanford University School of Medicine, Stanford, California, USA; 11 Kolling Institute of Medical Research, Sydney Medical School, University of Sydney, New South Wales, Australia; 12 Royal North Shore Hospital, St Leonards, New South Wales, Australia; 13 Janssen Research and Development, LLC, Spring House, Pennsylvania, USA

**Keywords:** anemia, biomarker, cardiovascular, kidney, mortality, SGLT2 inhibitor

## Abstract

**Introduction:**

Autoantibodies to erythropoietin receptor (anti-EPOR antibodies) have been identified in patients with various kidney diseases. However, data in patients with type 2 diabetes (T2D) and chronic kidney disease (CKD) is limited. We assessed the prevalence of anti-EPOR antibodies and their association with clinical outcomes in this population.

**Methods:**

The CREDENCE randomized patients with T2D and CKD to canagliflozin or placebo. Serum anti-EPOR antibodies, the exposure of interest, were measured using enzyme-linked immunosorbent assay. The primary outcome was doubling of serum creatinine, end-stage kidney disease, or death from kidney or cardiovascular (CV) causes. Secondary outcomes included CV and all-cause mortality. Multivariable Cox-regression models estimated associations between anti-EPOR antibodies and outcomes. The effects of canagliflozin on hemoglobin and hematocrit, stratified by the presence of anti-EPOR antibodies were assessed with a repeated measures mixed effects model.

**Results:**

Of 2600 participants with available biosamples, 191 (7.3%) were positive for anti-EPOR antibodies. Higher baseline anti-EPOR antibodies were associated with increased risk of primary outcome (hazard ratio [HR] per 1-SD increase = 1.12, 95% confidence interval [CI] = 1.01–1.24, *P* = 0.04), with CV death (HR = 1.27, 95% CI = 1.08–1.48, *P* < 0.01) and all-cause mortality (HR = 1.26, 95% CI = 1.11–1.43, *P* < 0.01). During follow-up, canagliflozin, compared to placebo, increased hemoglobin and hematocrit by 7.0 g/l (95% CI = 6.2–7.9) and 2.4% (2.2–2.7), respectively. These effects were consistent across patients with and without anti-EPOR antibodies (*P*-interaction = 0.24 and 0.36, respectively).

**Conclusion:**

In patients with T2D and CKD, anti-EPOR antibodies were associated with the composite kidney and CV outcome, as well as CV and all-cause mortality. Canagliflozin increased hemoglobin and hematocrit regardless of anti-EPOR antibodies.

## Graphical abstract

**Figure:**
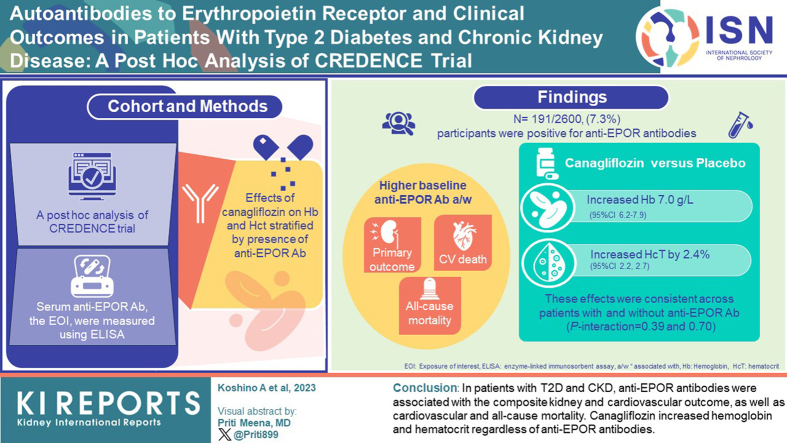

Patients with T2D and CKD are at high risk of kidney and CV events as well as premature death.^1^^,^^2^ Novel biomarkers can serve to screen patients at high risk of clinical adverse events.^3^ Previously, we identified anti-EPOR antibodies in patients with various kidney diseases, including diabetic kidney disease.^4^, ^5^, ^6^, ^7^ Although the pathogenic mechanisms underlying the development of autoantibodies in patients with T2D are unclear, anti-EPOR antibodies were associated with kidney disease progression in patients with diabetes.^5^^,^^7^ However, these studies were limited by low ethnic diversity, small sample sizes, case-control design, and lack of granular information on CV and mortality endpoints.^5^^,^^7^

Large clinical trials with sodium-glucose cotransporter 2 (SGLT2) inhibitors have shown that these agents reduce CKD progression, CV events, and extend survival in broad populations, including patients with CKD, irrespective of disease aetiology.^8^, ^9^, ^10^, ^11^, ^12^ *Post hoc* analyses of these trials reported that SGLT2 inhibitors, compared to placebo, increased hemoglobin and hematocrit and prevented anemia with one possible mechanism being enhanced erythropoietin (EPO) synthesis.^13^, ^14^, ^15^, ^16^, ^17^ Given that anti-EPOR antibodies were previously associated with resistance to erythropoiesis-stimulating agents,^18^ it is possible that the effect of SGLT2 inhibitors on anemia may be reduced in the presence of anti-EPOR antibodies.

This *post hoc* analysis of the CREDENCE trial assessed the association of anti-EPOR antibodies with composite kidney and CV events, and mortality in patients with T2D and CKD. We also examined whether the effects of canagliflozin on hemoglobin, hematocrit, and incident anemia were modified by anti-EPOR antibodies.

## Methods

### Participants and Study Design

CREDENCE was a double-blind, randomized, placebo-controlled trial conducted at 690 sites in 34 countries from March 2014 to May 2017 (ClinicalTrials.gov identifier: NCT02065791). The study protocol and results have been previously published.^11^ In brief, eligible participants were 30 years or older, had T2D, estimated glomerular filtration rate (eGFR) of 30 to <90 ml/min per 1.73 m^2^ and urine albumin-to-creatinine ratio (UACR) of >300 to 5000 mg/g. All participants were required to be receiving maximum tolerated or labelled dose of angiotensin-converting-enzyme inhibitor or angiotensin-receptor blocker for at least 4 weeks prior to randomization. Patients who had suspected type 1 diabetes or nondiabetic kidney disease were excluded. Participants were randomly assigned into either canagliflozin 100 mg daily or a matching placebo in a 1:1 manner. The trial protocol was approved by central and local ethics committees at each study site. All participants provided written informed consent. All volunteers were also offered the opportunity to join the exploratory biomarker evaluation, and those who agreed signed a separate consent form.

### Anti-EPOR Antibodies Measurement

We measured serum anti-EPOR antibodies at baseline and week 52 using an indirect enzyme-linked immunosorbent assay. The procedure was previously published.^6^^,^^7^ Briefly, recombinant human EPOR (R & D Systems, Minneapolis, MN) at a 5 μg/ml concentration in 0.2 M sodium bicarbonate buffer was coated onto 96-well microplates (R & D Systems, Minneapolis, MN). The remaining free binding sites were blocked using 1% bovine serum albumin in phosphate-buffered saline for 24 hours at 4 °C. After washing the microplates with Tween 20-Tris-buffered saline, samples at a 1000-fold dilution to 1% bovine serum albumin in phosphate-buffered saline were added to the microplates and incubated for 24 hours at 4 °C. Plates were washed 4 times with the same solution and incubated with goat anti-human Ig-conjugated with horseradish peroxidase (Sigma-Aldrich, Dorset, UK) at a 5000-fold dilution for 1 hour at room temperature. The substrate tetramethyl benzidine (KPL, Gaithersburg, MD) was added, and the reaction was stopped by adding stop solutions (KPL, Gaithersburg, MD). The optical density at 620 nm was measured by an automatic plate reader. The enzyme-linked immunosorbent assay unit (EU) of anti-EPOR antibodies was determined using a 3-point linear approximation of control serum (set as 10 EU at a 1000-fold dilution). In keeping with a previous study,^6^ we used the control serum collected from a Japanese patient with systemic lupus erythematosus and the cut-off of ≥2 EU for determining positivity. Measurements ≤0 EU were handled as 0.00001 EU for analysis purposes.

All measurements were carried out between July and November 2022 at the Department of Clinical Pharmacy and Pharmacology, University Medical Center Groningen, the Netherlands. Laboratory quality control was done using a Levely-Jennings chart plotting optical densities of control serum and the 1_3s_ and 2_2s_ rule of Westgard.^19^ Under this rule, if a control measurement in 1 plate deviates by more than 3 SDs or if measurements in 2 consecutive plates deviate by 2 to 3 SDs, samples on the plates are to be reassayed. Of 5091 samples, 215 samples (4.2%) were randomly selected for duplicate assessment. The average intraplate and interplate coefficients of variation were 6.8% and 9.3%, respectively.

### Outcomes

The prespecified primary outcome in the CREDENCE trial was a composite of doubling of serum creatine, end-stage kidney disease (maintenance dialysis, kidney transplantation, or a sustained eGFR of ≤15 ml/min per 1.73 m^2^) or death from kidney failure or CV disease.^11^ The secondary CREDENCE outcomes assessed in the current study were each component of the primary outcome (except for death from kidney failure, which only occurred in 5 patients), a renal-specific outcome (the primary outcome excluding CV death), hospitalization for heart failure, and all-cause death. All outcomes were adjudicated by independent blinded adjudication committees.^11^

Hemoglobin concentration and hematocrit level were measured at baseline and every 52 weeks. The measurements were carried out in a central laboratory. Anemia was defined as hemoglobin <130 g/l in men or <120 g/l in women, according to the World Health Organization guideline.^20^ We defined incident anemia as a hemoglobin value above this threshold among patients without anemia at baseline.

### Statistical Analysis

We summarized participant characteristics at baseline by the presence of anti-EPOR antibodies. Continuous variables were reported as mean (SD) or median (interquartile range), and categorical variables were reported as *n* (%). Baseline characteristics were compared between anti-EPOR-negative (<2 EU) and anti-EPOR-positive group (≥2 EU) using Student’s *t*-tests for approximately normally distributed continuous variables, Wilcoxon rank-sum tests for skewed variables and chi-square tests for categorical data. UACR and anti-EPOR antibody levels were natural log-transformed before analysis due to their skewness.

To assess the association between anti-EPOR antibodies at baseline and the primary and secondary outcomes, we made 3 multivariable adjusted Cox proportional hazards regression models with step-wise adjustment for selected baseline characteristics. In model 1, we adjusted for age, particiapnt reported biological sex (male or female), race, and randomized treatment. Model 2 was additionally adjusted for eGFR and log-transformed UACR. We further adjusted for history of CV disease and systolic blood pressure in model 3. We *a priori* selected these clinical characteristics as covariates for our Cox proportional hazards model because they were associated in prior studies with both anti-EPOR antibodies levels and adverse outcomes.^5^^,^^7^ HRs for anti-EPOR antibodies were calculated as both a continuous (per 1-SD increase of log-transformed anti-EPOR antibody levels) and a categorical variable (antibody-positive [≥2 EU] relative to antibody-negative [<2 EU]). To account for the competing risk of death in the association of anti-EPOR antibodies with the primary outcome and its components, sensitivity analyses using the Fine-Gray modification of the Cox model was performed. The association between anti-EPOR antibody levels and clinical outcomes with stratification by eGFR (30 to <45 ml/min per 1.73 m^2^, 45 to <60 ml/min per 1.73 m^2^, or 60 to <90 ml/min per 1.73 m^2^) and UACR categories (≤1000 mg/g or >1000 mg/g) was also assessed using model 3. The proportional hazards assumption was confirmed by testing the independence of scaled Schoenfeld residuals and time.

To evaluate the effect of canagliflozin on hemoglobin and hematocrit by anti-EPOR antibodies at baseline, least-square mean changes from baseline were calculated using linear mixed-effects models with a restricted maximum likelihood estimator. The models consist of the fixed categorical effects of randomized treatment, trial visit, eGFR at screening, and the interaction of treatment-by-visit plus the fixed continuous covariates of the baseline value and the baseline value-by-visit interaction. Patient-specific random effect was modeled using an unstructured covariance structure. The between-treatment-group differences in hemoglobin and hematocrit change during follow-up were calculated with the same model. To assess effect modification by anti-EPOR antibodies at baseline, we added main effect for anti-EPOR antibodies subgroup (positive or negative) and all 2-way and 3-way interaction terms between treatment, anti-EPOR antibodies and trial visit to the relevant models.

We estimated the effect of canagliflozin, compared to placebo, on time-to-incident anemia among patients without anemia at baseline by anti-EPOR antibodies. The Cox proportional hazard regression model was stratified by the eGFR categories at screening.^11^ The interaction was assessed by adding the treatment-by-anti-EPOR antibody subgroup interaction.

Finally, we described the proportion of anti-EPOR antibody positive patients by treatment group at baseline and week 52. Comparison between treatment groups was performed using a chi-square test. We also compared the change in antibody levels between treatment groups using analysis of covariance adjusted for randomized treatment and baseline antibody titer.

All statistical analysis was performed using R 4.1.3 (R Foundation for Statistical Computing, Vienna, Austria). All analyses were done by intention-to-treat.

## Results

### Baseline Characteristics

Out of 4401 CREDENCE participants, 2600 (59.1%) participants had available serum samples at baseline (Supplementary Figure S1). The characteristics of these 2600 individuals were generally comparable to the overall CREDENCE population.^11^ At baseline, the mean age was 63.3 (SD = 9.1) years, 33.7% of participants were female, 51.5% had a history of CV disease, 34.7% had anemia, and the mean hemoglobin was 132.7 (SD = 17.1) g/l (Table 1). The mean eGFR was 56.9 (SD = 18.3) ml/min per 1.73 m^2^, and the median UACR was 919.5 (interquartile range = 470.8–1760.8) mg/g. Of the participants, 191 (7.3%; 6.9% in canagliflozin group and 7.8% in placebo group) were positive (≥2 EU) for anti-EPOR antibodies (Table 1 and Supplementary Table S1). Patients with anti-EPOR antibodies, compared to those without, were older (64.6±9.0 vs. 63.2±9.1, *P* = 0.03) and more likely to have a history of CV disease (59.7% vs. 50.9%, *P* = 0.02). There was no difference in other characteristics, including prevalence of anemia, hemoglobin concentration, hematocrit, eGFR, UACR, and use of iron preparation and erythropoiesis-stimulating agents. The participants’ distribution by anti-EPOR antibodies titer are shown in Supplementary Figure S2. In the canagliflozin group, participant’s characteristics by randomized treatment was well-balanced between canagliflozin and placebo group and were similar to entire CREDENCE trial (Supplementary Table S1).

**Table 1:** Baseline characteristics of CREDENCE participants with antierythropoietin receptor antibodies measurements

Characteristics	Total *N* = 2600	Negative (<2.0 EU) *n* = 2409	Positive (≥2.0 EU) *n* = 191	*P*-value
Age, yrs	63.3±9.1	63.2±9.1	64.6±9.0	0.03
Female, *n* (%)	875 (33.7)	803 (33.3)	72 (37.7)	0.25
Race, *n* (%)				0.08
White	1866 (71.8)	1739 (72.2)	127 (66.5)	
Asian	332 (12.8)	310 (12.9)	22 (11.5)	
Black	143 (5.5)	129 (5.4)	14 (7.3)	
Other	259 (10.0)	231 (9.6)	28 (14.7)	
Current smoker, *n* (%)	381 (14.7)	352 (14.6)	29 (15.2)	0.91
History of CV disease, *n* (%)	1340 (51.5)	1226 (50.9)	114 (59.7)	0.02
Diabetes duration, years	16.0±8.7	15.9±8.6	17.0±10.0	0.10
BMI, kg/m^2^	31.9±6.3	31.9±6.2	31.4±6.7	0.28
Systolic BP, mm Hg	140.3±15.7	140.2±15.6	141.0±17.0	0.51
LDL cholesterol, mg/dl	95.1±41.0	95.1±41.3	95.4±37.4	0.91
Hemoglobin, g/L	132.7±17.1	132.8±17.0	132.0±18.1	0.58
Anemia, *n* (%)	839 (34.7)	770 (34.4)	69 (39.7)	0.18
Hematocrit, %	40.6±5.2	40.6±5.2	40.5±5.5	0.82
HbA1c, %	8.3±1.3	8.2±1.3	8.4±1.5	0.19
eGFR, ml/min per 1.73 m^2^	56.9±18.3	57.0±18.3	55.0±18.0	0.13
eGFR 30–45	728 (28.0)	669 (27.8)	59 (30.9)	0.50
eGFR 45–60	780 (30.0)	729 (30.3)	51 (26.7)	
eGFR 60–90	1092 (42.0)	1011 (42.0)	81 (42.4)	
UACR, mg/g	919.5 (470.8, 1760.8)	919.0 (469.0, 1771.0)	920.0 (490.5, 1644.0)	0.74
UACR > 1000	1195 (46.0)	1114 (46.2)	81 (42.4)	0.34
Canagliflozin group, *n* (%)	1313 (50.4)	1222 (50.7)	91 (47.6)	0.46
Anemia medication, *n* (%)				
Iron preparation	125 (4.8)	118 (4.9)	7 (3.7)	0.55
ESAs	17 (0.7)	17 (0.7)	0 (0.0)	0.49

BMI, body mass index; BP, blood pressure; CV, cardiovascular; eGFR, estimated glomerular filtration rate; ESAs, erythropoiesis-stimulating agents; HbA_1c_, glycated hemoglobin; IQR, interquartile range; LDL, low-density lipoprotein; UACR, urine albumin-to-creatinine ratio.

Continuous variables are reported as mean±SD, except for UACR shown as median (IQR) due to its skewness; categorical variables are reported as *n* (%). Anemia was defined as hemoglobin <130 g/l in men or <120 g/l in women.

### Associations of Anti-EPOR Antibodies With Clinical Outcomes

During a median follow-up of 2.8 years, 348 (13.4%) of participants experienced the primary composite outcome. The event rate for the primary outcome was higher in patients with anti-EPOR antibodies compared to those without anti-EPOR antibodies (63.0 vs. 50.1 per 1000 patient-years; Table 2). After adjusting for age, sex, race, and randomized treatment, anti-EPOR antibodies (as a continuous variable) were significantly associated with the primary outcome (model 1). Anti-EPOR antibodies remained significant after adjusting for kidney disease parameters alone (eGFR and log-transformed UACR - model 2), or for both kidney and CV parameters (eGFR, log-transformed UACR, history of CV disease and systolic blood pressure - model 3). In model 3, higher log-transformed anti-EPOR antibodies (per 1-SD increase) at baseline was associated with increased risk of the primary outcome (HR = 1.12, 95% CI = 1.01– 1.24, *P* = 0.04; Table 2). Anti-EPOR antibodies (fitted as a categorical variable) were not associated with the primary outcome (Table 2). There was no association of anti-EPOR antibodies with the renal-specific composite of doubling of serum creatinine, end-stage kidney disease or renal death, or with hospitalization for heart failure (Table 2 and Supplementary Table S2). These findings remained unchanged when the competing risk of non-CV, nonrenal, or all-cause death were taken into account (Supplementary Table S3).

**Table 2:** Associations of antierythropoietin receptor antibodies at baseline with primary composite outcome, renal specific composite outcome, and mortality outcomes

Outcome	Events		Model 1		Model 2		Model 3	
	*n* (%)	/ 1000 PY	HR (95% CI)	*P*-value	HR (95% CI)	*P*-value	HR (95% CI)	*P*-value
Primary composite outcome: doubling of serum creatinine, ESKD, renal death, or CV death								
Per 1-SD increase			1.14 (1.02–1.26)	0.02	1.12 (1.01–1.24)	0.03	1.12 (1.01–1.24)	0.04
Negative (*N* = 2409)	318 (13.2)	50.1	Reference		Reference		Reference	
Positive (*N* = 191)	30 (15.7)	63.0	1.27 (0.87–1.85)	0.21	1.25 (0.86–1.83)	0.24	1.25 (0.86–1.82)	0.25
Renal specific composite outcome: doubling of serum creatinine, ESKD, or renal death								
Per 1-SD increase			1.07 (0.94–1.23)	0.29	1.07 (0.93–1.22)	0.34	1.06 (0.93–1.22)	0.36
Negative	208 (8.6)	32.8	Reference		Reference		Reference	
Positive	18 (9.4)	37.9	1.20 (0.74–1.95)	0.45	1.21 (0.75–1.97)	0.43	1.25 (0.77–2.03)	0.37
CV death								
Per 1-SD increase			1.30 (1.11–1.52)	<0.01	1.28 (1.10–1.50)	<0.01	1.27 (1.08–1.48)	<0.01
Negative	130 (5.4)	20.0	Reference		Reference		Reference	
Positive	16 (8.4)	32.7	1.57 (0.93–2.65)	0.09	1.54 (0.91–2.60)	0.11	1.47 (0.87–2.47)	0.15
Death from any cause								
Per 1-SD increase			1.28 (1.13–1.45)	<0.01	1.27 (1.12–1.44)	<0.01	1.26 (1.11–1.43)	<0.01
Negative	198 (8.2)	30.4	Reference		Reference		Reference	
Positive	29 (15.2)	59.3	1.85 (1.25–2.73)	<0.01	1.82 (1.23–2.70)	<0.01	1.76 (1.19–2.60)	<0.01

CI, confidence interval; CV, cardiovascular; eGFR, estimated glomerular filtration rate; ESKD, end-stage kidney disease; HR, hazard ratio; PY, person-year; UACR, urine albumin-to-creatinine ratio.

Models were adjusted for the following covariates. Model 1: age, sex, race, and randomized treatment. Model 2: covariates of model 1 + eGFR and log-transformed UACR. Model 3: covariates of model 2 + history of CV disease and systolic blood pressure.

**Figure 1:**
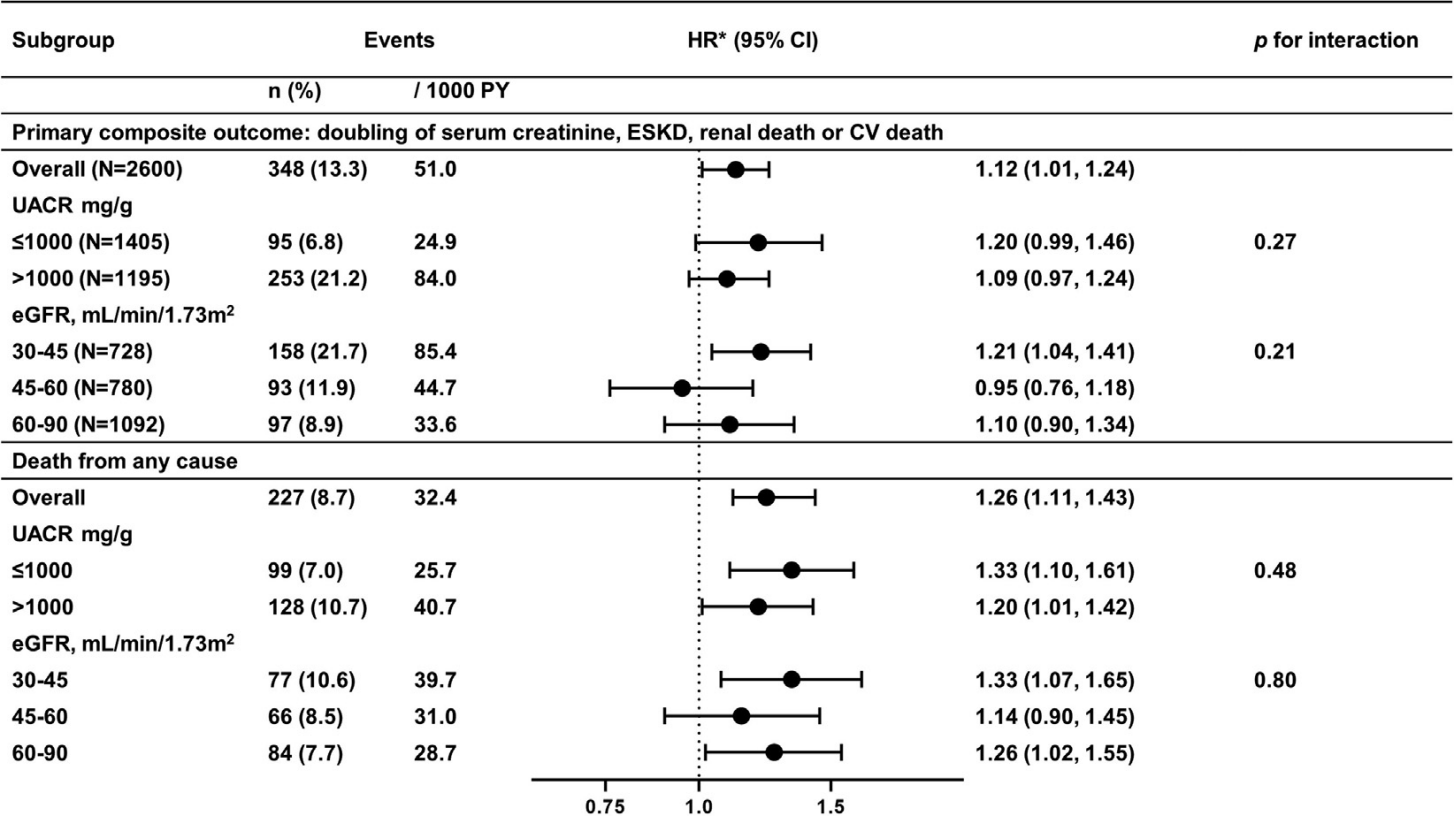
Associations of antierythropoietin receptor antibodies with the primary composite outcome and all-cause mortality by baseline UACR and eGFR categories. ∗Per 1-SD increase of log-transformed anti-EPOR antibodies. The Cox proportional hazard model was adjusted for the following covariates: age, sex, race, randomized treatment, history of cardiovascular disease, systolic blood pressure, eGFR, and log-transformed UACR. BP, blood pressure; eGFR, estimated glomerular filtration rate; EPOR, erythropoietin receptor; HR, hazard ratio; PY, person-year; UACR, urine albumin-to-creatinine ratio.

Event rates for CV and all-cause death were higher in patients with versus without anti-EPOR antibodies (Table 2). In fully adjusted Cox proportional hazards models (Table 2**,** model 3), higher anti-EPOR antibodies at baseline were associated with both an increased risk of CV death (HR = 1.27, 95% CI = 1.08–1.48, *P <* 0.01) and all-cause death (HR = 1.26, 95% CI = 1.11–1.43, *P <* 0.01). The associations of anti-EPOR antibodies with the primary outcome and all-cause death were consistent across eGFR and UACR categories (Figure 1).

### Effect of Canagliflozin on Anemia by Anti-EPOR Antibodies

**Figure 2:**
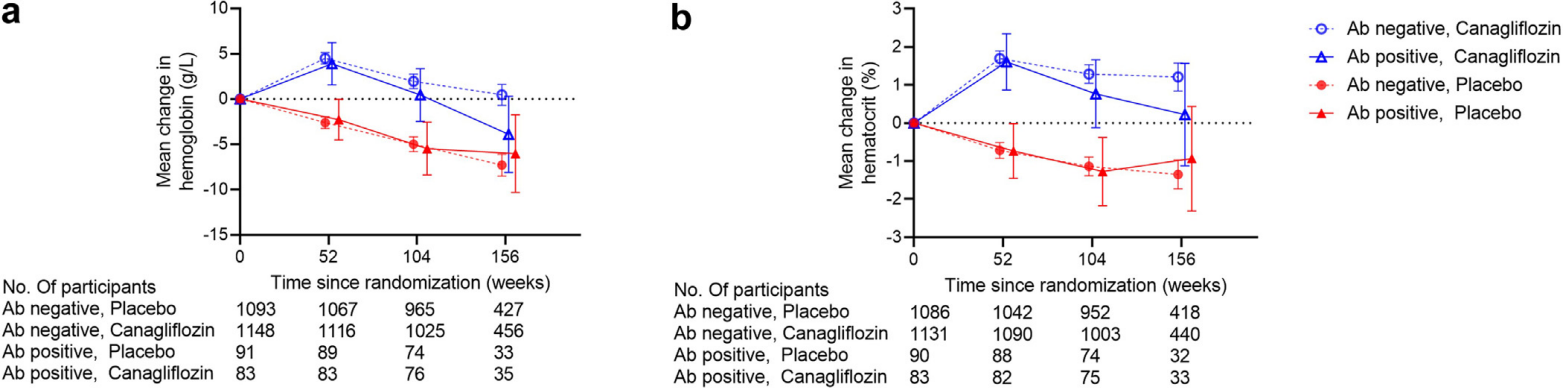
Effect of canagliflozin on (a) hemoglobin and (b) hematocrit over time by antierythropoietin receptor antibodies at baseline. Linear mixed-effects models with a restricted maximum likelihood-based repeated measures calculated the least-squares mean changes from baseline in hemoglobin and hematocrit. The model included fixed, categorical effects of therapy, trial visit, eGFR at screening, and treatment-by-visit interaction fixed along with fixed, continuous baseline value variables and baseline value by visit interaction. For anti-EPOR antibodies subgroup (positive or negative), we added main effect for the subgroup and all 2-way and 3-way interaction terms between treatment, anti-EPOR antibodies, and trial visit to the models. An unstructured covariance structure was used to model the within-patient errors. eGFR, estimated glomerular filtration rate; EPOR, erythropoietin receptor.

Hemoglobin and hematocrit increased in the canagliflozin group from baseline to week 52 (Figure 2, Supplementary Table S4). In contrast, hemoglobin and hematocrit in the placebo group decreased over time. The mean difference during follow-up between the canagliflozin group and the placebo group was 7.0 g/l (95% CI = 6.2–7.9) for hemoglobin and 2.4% (95% CI = 2.2–2.7) for hematocrit, respectively. These effects of canagliflozin on hemoglobin and hematocrit were consistent across patients with and without anti-EPOR antibodies (*P* for interaction = 0.24 and 0.36, respectively; Figure 2, Supplementary Table S4).

Among patients without anemia at baseline, canagliflozin reduced the risk of incident anemia (96.7 vs. 176.2 per 1000 person-year; HR = 0.51, 95% CI = 0.42–0.61, *P<*0.01; Table 3). The reduction in risk of anemia with canagliflozin was not modified by anti-EPOR antibodies at baseline (*P* for interaction = 0.27).

**Table 3:** Effects of canagliflozin on time to incident anemia by antierythropoietin receptor antibodies at baseline

Subgroups	Events /1000PY		HR (95%CI)	*P* for interaction
	Canagliflozin	Placebo		
Overall (*N* = 1530)	96.7	176.2	0.51 (0.42–0.61)	
Negative (*n* = 1427)	97.9	173.7	0.52 (0.43–0.64)	0.27
Positive (*n* = 103)	82.1	216.1	0.33 (0.15–0.69)	

CI, confidence interval; HR, hazard ratio; PY, person-year.

Cox proportional hazard regression model was stratified with prespecified eGFR strata at screening.

Antierythropoietin receptor antibodies ≥2 EU were considered positive. Anemia was defined as hemoglobin <130 g/l in men or <120 g/l in women.

### Effect of Canagliflozin on Anti-EPOR Antibodies

Fifty-two weeks after randomization, the proportion of patients with anti-EPOR antibodies was similar between the canagliflozin group (7.6%) and placebo group (6.5 %; *P* = 0.29; Supplementary Table S5). There was also no difference in the change of antibody titer from baseline to week 52 between the canagliflozin and placebo group (between group difference: −0.1 EU [−17.3 to 20.7], *P* = 0.90).

## Discussion

In this *post hoc* analysis of the CREDENCE trial, anti-EPOR antibodies were detected in 7.3 % of patients with T2D and CKD. Higher anti-EPOR antibodies were associated with the primary composite outcome, CV death and all-cause mortality. The beneficial effect of canagliflozin on anemia was consistent across patients with and without anti-EPOR antibodies.

Previously, a nested case-control study of a randomized controlled trial reported that anti-EPOR antibodies improved the prediction of kidney disease progression independent of traditional risk factors in patients with T2D at high CV risk.^7^ The current data from the CREDENCE trial provides new evidence on the association of anti-EPOR antibodies with CV and kidney events in a population with T2D and more severe kidney disease. In contrast to previous studies,^5^^,^^7^ the association of anti-EPOR antibodies with the primary composite outcome was mainly driven by the association with the risk of CV death. The difference between the current and previous studies may be attributed to the differences in patient characteristics, differences in study design (nested case-control vs. more robust observational cohort) and differences in analytical methods, including time-to-event analysis and adjusting for competing risks in the current study. Higher anti-EPOR antibodies were also associated with increased risk of CV and all-cause mortality. Risk assessment for death has a substantial clinical relevance, especially in elderly patients with diabetes and CKD. In a longitudinal community-based cohort study, patients aged 65 or more with CKD were 6 and 13 times more likely to die from CV and any-cause before developing end-stage kidney disease.^1^ Therefore, although further validation studies are necessary, anti-EPOR antibodies might aid in identification of individuals with T2D and CKD at high risk of mortality.

SGLT2 inhibitor treatment increased hemoglobin and hematocrit, and prevented anemia compared to placebo in several large clinical trials, including the CREDENCE trial.^13^, ^14^, ^15^ Statistical mediation analyses of these trials have reported a strong association between hemoglobin change with SGLT2 inhibition and CV and kidney protection.^21^, ^22^, ^23^ There is evidence that SGLT2 inhibitors transiently increased EPO levels and this could explain at least part of the beneficial effect observed for anemia.^16^^,^^17^ Previously, we reported that addition of anti-EPOR antibodies hampered EPO-dependent proliferation of erythroid progenitor cells.^6^ Despite this possible blockade of the EPO-EPOR system in bone marrow by anti-EPOR antibodies, we found consistent beneficial effects of SGLT2 inhibitors on hemoglobin, hematocrit, and incident anemia in anti-EPOR antibodies positive and negative individuals, suggesting that anti-EPOR antibodies do not alter observed beneficial effects of canagliflozin on fluid volume and iron homeostasis.^14^^,^^16^^,^^24^^,^^25^

In contrast to previous data from Japanese patients,^5^ hemoglobin and hematocrit were similar between patients with and without anti-EPOR antibodies in the current study. EPOR protein forms a homodimer on erythroid progenitor cells and enhances differentiation into erythrocytes. EPOR also exists on organ-specific cells such as cardiomyocytes and glomerular and tubular epithelial cells but as a complex of EPOR protein and β common chain.^26^^,^^27^ A non-erythropoietic organ protective effect of EPO via this complex receptor has been reported in experimental studies, although this has not been proven in clinical trials.^26^^,^^27^ Previously, we reported that IgG fractions containing anti-EPOR antibodies inhibit the antiinflammatory effect of EPO in kidney tubular epithelial cells.^5^ In addition, some studies reported heterogeneity among individuals in the autoantibody binding sites and their effect.^28^^,^^29^ The role of EPO-EPOR in the pathophysiology of anemia may differ by ethnicity. Because the proportion of participants from Japan was small in the CREDENCE trial, this may explain potential differences with earlier studies, which were exclusively performed in Japanese participants. Further studies are needed to assess whether anti-EPOR antibodies are on the causal pathway to adverse outcomes, including anemia, or are simply a marker of poor prognosis.

Key strengths of this study include its multinational population with T2D and CKD, and rigorously adjudicated clinical outcomes. The study has some limitations. First, the association between anti-EPOR antibodies and the primary outcome was relatively small (HR per 1-SD increase 1.12, 95% CI = 1.01–1.24, *P* = 0.04). However, the significant association after adjustment of multiple risk factors suggests that anti-EPOR antibodies could be a relevant prognostic biomarker beyond clinically available risk factors in patients with T2D. Second, the *post hoc* study design increases the possibility of chance findings. Third, although we adjusted for multiple confounders in the analyses of associations of anti-EPOR antibodies with clinical outcomes, remaining confounding cannot be ruled out. For example, we were unable to take regional information into account due to the privacy regulations. Fourth, the number of patients in the anti-EPOR antibodies positive (≥2 EU) group was relatively small. This might limit the statistical power to detect the association between EPOR antibodies fitted as categorical variable and clinical outcomes. Another limitation is that EPO concentration data were unavailable in the current study. It is possible that EPO production is stimulated in patients with anti-EPOR antibodies, which might explain the similar hemoglobin and hematocrit levels between patients with and without anti-EPOR antibodies, though this hypothesis could not be tested.

In summary, we demonstrated that anti-EPOR antibodies were associated with an increased risk of the primary composite outcome and mortality in patients with T2D and CKD. The beneficial effect of canagliflozin on anemia was consistent across patients with and without anti-EPOR antibodies. Further studies are necessary to better understand the pathophysiology of anti-EPOR antibodies and to validate their use in clinical practice.

## Disclosure

AK and AH declared no conflicting interests. BLN has received fees for advisory boards, scientific presentations, speaker fees, steering committee roles and travel support from the American Diabetes Association, AstraZeneca, Bayer, Boehringer and Ingelheim, Cambridge Healthcare Research, Janssen and Medscape, with all honoraria paid to his institution. MO reports serving on the speakers bureau for Daiichi Sankyo, Japan, and Mitsubishi Tanabe Pharma. TT reports receiving the speaker fees and research funding from Mitsubishi Tanabe Pharma and AstraZeneca. CA is supported by an NHMRC/Medical Research Future Fund Priority Investigator Grant and a New South Wales Health Early to Mid-Career Research Grant and is an employee of the George Institute for Global Health. She is responsible for the secondary analysis program for the CANVAS Program and CREDENCE trial. She has received honoraria from AstraZeneca and Amgen. BN is supported by a National Health and Medical Research Council Investigator Grant (APP1197709). His institution has received grants, travel, and consulting fees from Janssen pharmaceuticals for work done on CANVAS and CREDENCE. MJ is responsible for research projects that have received funding from Amgen, Baxter, CSL, Dimerix, Eli Lilly, Gambro, and MSD; and has received advisory, steering committee and/or speaker fees from Akebia, Amgen, Astra Zeneca, Baxter, Bayer, Boehringer Ingelheim, Chinook, CSL, Janssen, MSD, Roche, and Vifor Pharma, with any consultancy, honoraria, or travel support paid to her institution. SVB has received speaking fees from Bayer, Pfizer, and Vifor Pharma; has attended advisory boards for AstraZeneca, Bayer, and Vifor Pharma; and has received nonfinancial research support from Bayer. All honoraria received were paid to SVB’s institution. KWM has received research support from Afferent; Amgen; Apple, Inc.; AstraZeneca; Cardiva Medical, Inc.; Daiichi; Ferring; Google (Verily); Johnson & Johnson; Luitpold; Medtronic; Merck; National Institutes of Health; Novartis; Sanofi; St Jude; and Tenax. He also has served as a consultant (speaker fees for continuing medical education events only) for Abbott, Ablynx, AstraZeneca, Baim Institute, Boehringer Ingelheim, Bristol-Myers Squibb, Elsevier, GlaxoSmithKline, 10.13039/100004331Johnson & Johnson, MedErgy, Medscape, Mitsubishi, Myokardia, National Institutes of Health, Novartis, Novo Nordisk, Portola, Radiometer, Regeneron, Springer Publishing, and University of California San Francisco. CP has served on the steering committee for CREDENCE, has been a speaker for Janssen Cilag, an advisory board member and speaker for Astra Zeneca, and a speaker for Eli Lilly and Boehringer Ingelheim. MKH is an employee of Janssen Research & Development, LLC. TW is supported by the Japan Society for the Promotion of Science Grants-in-Aid for Scientific Research [Grant Number: 20KK0191]. HJLH reports ongoing consultancy agreements with AstraZeneca, Bayer, Boehringer Ingelheim, Chinook, CSL Behring, Dimerix, Eli-Lilly, Gilead, Janssen, Merck, Novartis, Novo Nordisk, and Travere Pharmaceuticals; has served as a consultant for AbbVie, AstraZeneca, Bayer, Boehringer Ingelheim, Chinook, CSL Behring, Dimerix, Eli-Lilly Gilead, Janssen, Mitsubishi Tanabe, Mundipharma, Novartis, Novo Nordisk, and Travere Pharmaceuticals; reports research funding from AbbVie, AstraZeneca, Boehringer Ingelheim, Janssen research support (grant funding directed to employer), and Novo Nordisk; receives lecture fees from AstraZeneca and Novo Nordisk; and was a member on the speakers bureau for AstraZeneca.

## Supplementary Material

Supplementary File (PDF)

**Figure S1.** CONSORT diagram describing the number of participants included in each analysis.

**Figure S2.** Patients’ distribution by anti-EPOR antibody titer at baseline.

**Table S1.** Baseline characteristics of CREDENCE participants with antierythropoietin receptor antibodies by treatment allocation and those in the entire trial.

**Table S2.** Associations of antierythropoietin receptor antibodies at baseline with components of primary composite outcome and hospitalization due to heart failure.

**Table S3.** Associations of antierythropoietin receptor antibodies at baseline with kidney and cardiovascular events taking competing risk of death into account.

**Table S4.** Effects of canagliflozin on hemoglobin and hematocrit by antierythropoietin receptor antibodies at baseline.

**Table S5.** Proportion of patients with antierythropoietin receptor antibodies at baseline and week 52, by treatment group.

**CONSORT checklist.**
