# Supplementary material for: Autoantibodies to Erythropoietin Receptor and Clinical Outcomes in Patients With Type 2 Diabetes and CKD: A Post Hoc Analysis of CREDENCE Trial
Source: Kidney Int Rep. 2023 Dec 1;9(2):347–55. doi: 10.1016/j.ekir.2023.11.024 (PMC10850998; doi:10.1016/j.ekir.2023.11.024)
Supplement: Supplementary File (PDF) [file mmc1.pdf]

Supplementary information

Table S1: Baseline characteristics of CREDENCE participants with anti-erythropoietin receptor antibodies by treatment allocation and those in the entire trial

	Patients with anti-EPOR antibodies measurement			Overall CREDENCE trial
	Total	Canagliflozin	Placebo	
	N=2600	N=1313	N=1287	N=4401
Age, years	63.3±9.1	63.2±9.1	63.4±9.2	63.0±9.2
Female, n (%)	875 (33.7)	449 (34.2)	426 (33.1)	1494 (33.9)
Race, n (%)				
White	1,866 (71.8)	952 (72.5)	914 (71.0)	2,931 (66.6)
Asian	332 (12.8)	160 (12.2)	172 (13.4)	877 (19.9)
Black	143 (5.5)	73 (5.6)	70 (5.4)	224 (5.1)
Other	259 (10.0)	128 (9.7)	131 (10.2)	369 (8.4)
Current smoker, n (%)	381 (14.7)	195 (14.9)	186 (14.5)	639 (14.5)
History of CV disease, n (%)	1,340 (51.5)	678 (51.6)	662 (51.4)	2,220 (50.4)
Diabetes duration, years	16.0±8.7	15.8±8.8	16.2±8.6	15.8±8.6
BMI, kg/m^2	31.9±6.3	31.9±6.3	31.8±6.2	31.3±6.2
Systolic BP, mmHg	140.3±15.7	140.3±15.9	140.2±15.6	140.0±15.6
LDL cholesterol, mg/dL	95.1±41.0	95.7±43.0	94.5±39.0	96.4±41.3
Hemoglobin, g/L	132.7±17.1	133.3±17.3	132.2±16.8	132.2±17.8
Anemia, n (%)	839 (34.7)	409 (33.2)	30 (36.3)	447 (35.7)
Hematocrit, %	40.6±5.2	40.8±5.1	40.5±5.2	40.6±5.3
HbA1c, %	8.3±1.3	8.3±1.3	8.2±1.3	8.3±1.3
eGFR, mL/min/1.73m^2	56.9±18.3	56.8±18.2	57.0±18.3	56.2±18.2
eGFR30-45	728 (28.0)	379 (28.9)	49 (27.1)	315 (29.9)
eGFR45-60	780 (30.0)	386 (29.4)	94 (30.6)	280 (29.1)
eGFR60-90	1,092 (42.0)	548 (41.7)	544 (42.3)	806 (41.0)
UACR, mg/g	919.5 (470.8, 1760.8)	928.0 (471.0, 1766.0)	893.0 (47000, 1743.5)	927.0 (463.0, 1833.0)
UACR>1000	1195 (46.0)	604 (46.0)	591 (45.9)	2053 (46.6)
Anemia medication, n (%)				
Iron preparation	125 (4.8)	65 (5.0)	60 (4.7)	233 (5.3)
ESAs	17 (0.7)	9 (0.7)	8 (0.6)	33 (0.7)
Positive for anti-EPOR antibodies	191 (7.3)	91 (6.9)	100 (7.8)	

Continuous variables are reported as mean±SD, except for UACR shown as median (IQR) due to its skewness; categorical variables are reported as n (%). Anemia was defined as hemoglobin <130 g/L in men or <120 g/L in women.

BMI, body mass index; BP, blood pressure; CV, cardiovascular; eGFR, estimated glomerular filtration rate; ESAs, erythropoiesis-stimulating agents; HbA_{1c}, glycated hemoglobin; UACR, urine albumin-creatinine ratio

Table S2. Associations of anti-erythropoietin receptor antibodies at baseline with components of primary composite outcome and hospitalization due to heart failure

Outcome	Events		Model 1		Model 2		Model 3	
	**n (%)**	**/ 1000 PY**	**HR (95% CI)**	***p* value**	**HR (95% CI)**	***p* value**	**HR (95% CI)**	***p* value**
**Doubling of serum creatinine**								
Per 1-SD increase			1.02 (0.88, 1.19)	0.78	1.02 (0.87, 1.18)	0.83	1.01 (0.87, 1.18)	0.88
Negative	170 (7.1)	26.7	Reference		Reference		Reference	
Positive	13 (6.8)	27.3	1.07 (0.61, 1.89)	0.81	1.10 (0.62, 1.94)	0.74	1.13 (0.64, 2.00)	0.67
**ESKD**								
Per 1-SD increase			1.10 (0.94, 1.29)	0.23	1.09 (0.93, 1.27)	0.31	1.09 (0.93, 1.27)	0.30
Negative	146 (6.1)	22.8	Reference		Reference		Reference	
Positive	13 (6.8)	27.2	1.23 (0.70, 2.17)	0.48	1.23 (0.70, 2.19)	0.47	1.32 (0.74, 2.34)	0.35
**Hospitalization due to heart failure**								
Per 1-SD increase			0.92 (0.77, 1.10)	0.37	0.90 (0.75, 1.08)	0.26	0.89 (0.75, 1.07)	0.23
Negative	117 (4.9)	18.4	Reference		Reference		Reference	
Positive	14 (7.3)	29.7	1.59 (0.91, 2.77)	0.10	1.56 (0.89, 2.72)	0.12	1.50 (0.86, 2.62)	0.15

Models were adjusted for the following covariates. Model 1: age, sex, race, and randomized treatment. Model 2: covariates of model 1 + estimated glomerular filtration rate and log transformed urine albumin-creatinine ratio. Model 3: covariates of model 2 + history of CV disease and systolic blood pressure.
Four and one renal death occurred in the anti-EPOR antibodies negative and positive groups, respectively. Because of the small number of events, Cox models are not applied for renal death.
CV, cardiovascular; ESKD, end-stage kidney disease; HR, hazard ratio; PY, person-year

Table S3. Associations of anti-erythropoietin receptor antibodies at baseline with kidney and cardiovascular events taking competing risk of death into account

Outcome	Events		Model 1		Model 2		Model 3	
	n (%)	/ 1000 PY	sHR (95% CI)	*p* value	sHR (95% CI)	*p* value	sHR (95% CI)	*p* value
**Primary composite outcome: doubling of serum creatinine, ESKD, renal death or CV death**								
Per 1-SD increase			1.13 (1.02, 1.26)	0.02	1.11 (1.00, 1.24)	0.04	1.11 (1.00, 1.23)	0.05
Negative (N=2409)	318 (13.2)	50.1	Reference		Reference		Reference	
Positive (N=191)	30 (15.7)	63.0	1.24 (0.85, 1.80)	0.27	1.23 (0.84, 1.79)	0.29	1.22 (0.84, 1.78)	0.30
**Renal specific composite outcome: doubling of serum creatinine, ESKD or renal death**								
Per 1-SD increase			1.07 (0.94, 1.23)	0.29	1.07 (0.93, 1.22)	0.34	1.06 (0.93, 1.22)	0.36
Negative	208 (8.6)	32.8	Reference		Reference		Reference	
Positive	18 (9.4)	37.9	1.20 (0.74, 1.95)	0.45	1.21 (0.75, 1.97)	0.43	1.25 (0.77, 2.03)	0.37
**CV death**								
Per 1-SD increase			1.30 (1.11, 1.52)	<0.01	1.28 (1.10, 1.50)	<0.01	1.27 (1.08, 1.48)	<0.01
Negative	130 (5.4)	20.0	Reference		Reference		Reference	
Positive	16 (8.4)	32.7	1.57 (0.93, 2.65)	0.09	1.54 (0.91, 2.60)	0.11	1.47 (0.87, 2.47)	0.15
**Hospitalization due to heart failure**								
Per 1-SD increase			0.92 (0.77, 1.10)	0.37	0.90 (0.75, 1.08)	0.26	0.89 (0.75, 1.07)	0.23
Negative	117 (4.9)	18.4	Reference		Reference		Reference	
Positive	14 (7.3)	29.7	1.59 (0.91, 2.77)	0.1	1.56 (0.89, 2.72)	0.12	1.50 (0.86, 2.62)	0.15

Fine and Gray models were adjusted for the following covariates. Model 1: age, sex, race, and randomized treatment. Model 2: covariates of model 1 + estimated glomerular filtration rate and log transformed urine albumin-creatinine ratio. Model 3: covariates of model 2 + history of CV disease and systolic blood pressure.
CV, cardiovascular; ESKD, end-stage kidney disease; sHR, sub-distribution hazard ratio; PY, person-year

Table S4. Effects of canagliflozin on hemoglobin and hematocrit by anti-erythropoietin receptor antibodies at baseline

	Mean at baseline (SD)		Mean difference during follow-up (95%CI) *	*p* for interaction
	Canagliflozin	Placebo		
Hemoglobin concentration, g/L				
Overall (N=2415)	133.3 (17.3)	132.2 (16.8)	7.0 (6.2, 7.9)	
Negative (N=2241)	133.1 (17.2)	132.4 (16.8)	7.1 (6.3, 8.0)	0.24
Positive (N=174)	135.8 (18.6)	128.6 (16.9)	5.4 (2.2, 8.6)	
Hematocrit concentration, %				
Overall (N=2390)	40.8 (5.3)	40.4 (5.1)	2.4 (2.2, 2.7)	
Negative (N=2217)	40.7 (5.3)	40.5 (5.1)	2.4 (1.1, 3.0)	0.36
Positive (N=173)	41.8 (5.9)	39.4 (4.8)	2.0 (1.1, 3.2)	

Linear mixed-effects models with a restricted maximum likelihood-based repeated measures calculated the least-squares mean changes from baseline in hemoglobin and hematocrit. The model included fixed, categorical effects of therapy, trial visit, eGFR at screening, and treatment-by-visit interaction fixed along with fixed, continuous baseline value variables and baseline value by visit interaction. For anti-EPOR antibodies subgroup (positive or negative), we added main effect for the subgroup and all two-way and three-way interaction terms between treatment, anti-EPOR antibodies and trial visit to the models. An unstructured covariance structure was used to model the within-patient errors.
*Canagliflozin group minus placebo group

Table S5. Proportion of patients with anti-erythropoietin receptor antibodies at baseline and week 52, by treatment group

	Canagliflozin	Placebo	*p* value
Ab positive at baseline, n(%)	91 (6.9)	100 (7.8)	
Ab positive at week 52, n(%)	90 (7.6)	74 (6.5)	0.29

The number of participants was: 1313 in canagliflozin and 1287 in placebo group at baseline; 1182 in canagliflozin and 1140 in placebo at week 52. P value was calculated using the chi-square test to compare the proportion of erythropoietin receptor antibody-positive (≥2 EU) patients in the canagliflozin and placebo groups at week 52.

FigureS1. CONSORT diagram describing the number of participants included in each analysis

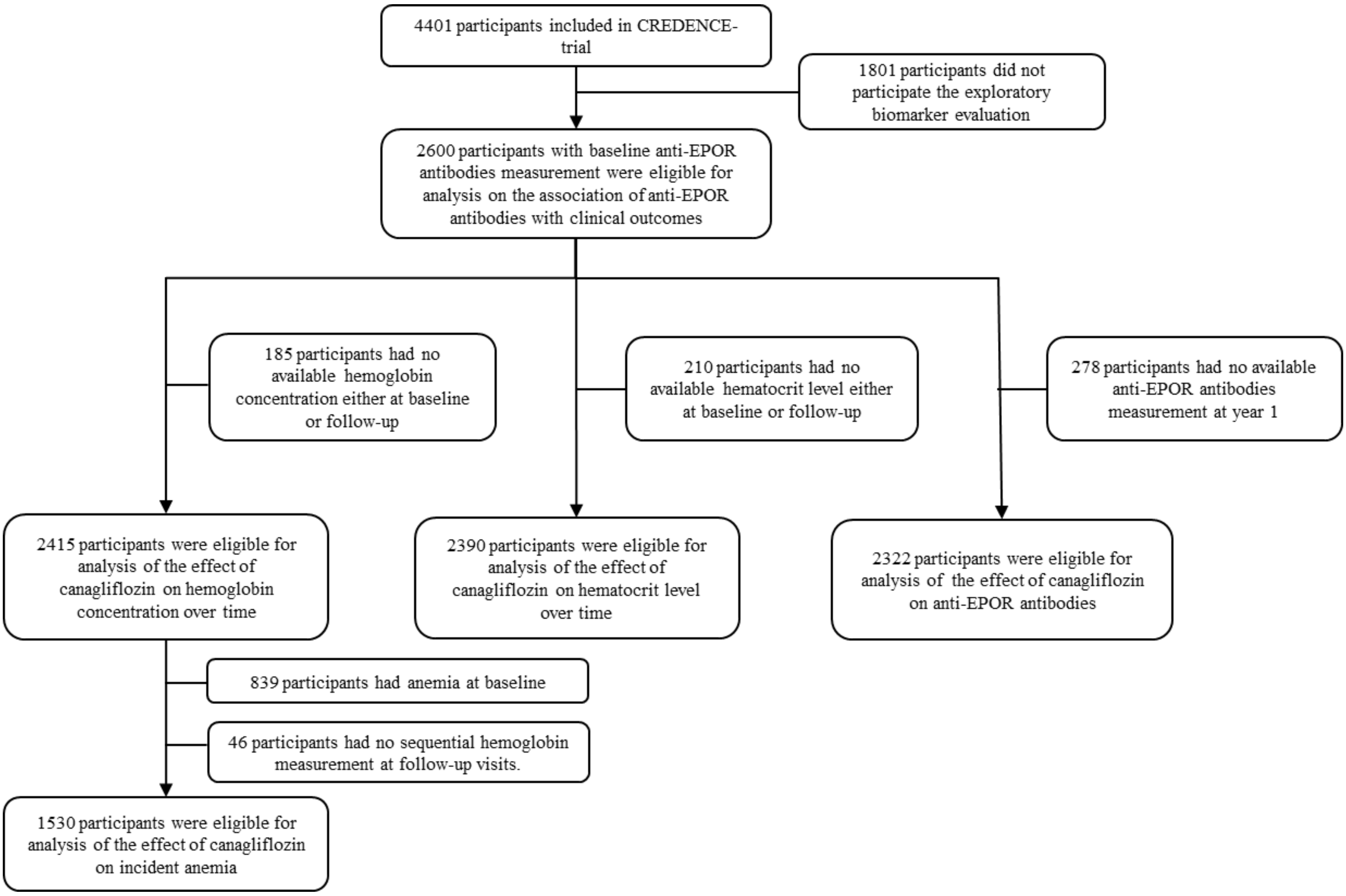

Anemia was defined as hemoglobin <130 g/L in men or <120 g/L in women.
EPOR; erythropoietin receptor

FigureS2. Patients' distribution by anti-EPOR antibody titer at baseline

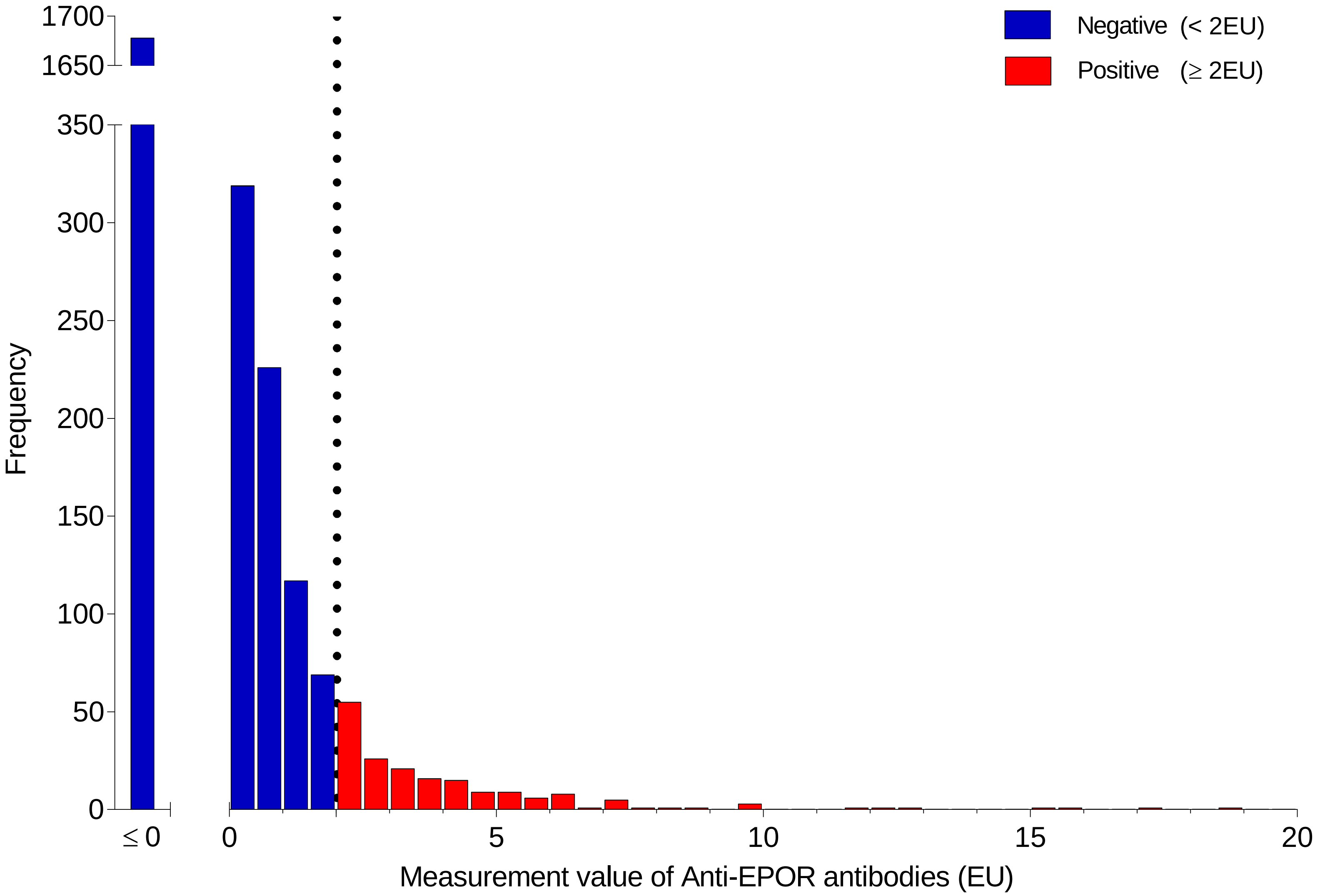

There were 7 participants with anti-EPOR antibodies of >20EU.

CONSORT 2010 checklist of information to include when reporting a randomised trial*

Section/Topic	Item No	Checklist item	Reported on page No
**Title and abstract**			
	1a	Identification as a randomised trial in the title	Page 1
	1b	Structured summary of trial design, methods, results, and conclusions (for specific guidance see CONSORT for abstracts)	Page 3, structured per journal requirements
**Introduction**			
Background and objectives	2a	Scientific background and explanation of rationale	Page 4
	2b	Specific objectives or hypotheses	Page 4
**Methods**			
Trial design	3a	Description of trial design (such as parallel, factorial) including allocation ratio	Pages 4 and 5; also included in the primary publication which is referenced (reference 11)
	3b	Important changes to methods after trial commencement (such as eligibility criteria), with reasons	Included in primary publication which is referenced (reference 11)
Participants	4a	Eligibility criteria for participants	Pages 4 and 5; also included in the primary publication which is referenced (reference 11)
	4b	Settings and locations where the data were collected	Included in primary publication which is referenced (reference 11).<br>Geographical information for the biomarker cohort was not provided due to privacy regulations.
Interventions	5	The interventions for each group with sufficient details to allow replication, including how and when they were actually administered	Pages 4 and 5; also included in the primary publication which is referenced (reference 16)
Outcomes	6a	Completely defined pre-specified primary and secondary outcome measures, including how and when they were assessed	Page 6 (outcomes assessed in this post hoc analysis)
	6b	Any changes to trial outcomes after the trial commenced, with reasons	Not applicable

Sample size	7a	How sample size was determined	Included in primary publication which is referenced (reference 11).
	7b	When applicable, explanation of any interim analyses and stopping guidelines	Included in primary publication which is referenced (reference 11).
Randomisation:			
Sequence generation	8a	Method used to generate the random allocation sequence	Included in primary publication which is referenced (reference 11).
	8b	Type of randomisation; details of any restriction (such as blocking and block size)	Included in primary publication which is referenced (reference 11).
Allocation concealment mechanism	9	Mechanism used to implement the random allocation sequence (such as sequentially numbered containers), describing any steps taken to conceal the sequence until interventions were assigned	Included in primary publication which is referenced (reference 11).
Implementation	10	Who generated the random allocation sequence, who enrolled participants, and who assigned participants to interventions	Included in primary publication which is referenced (reference 11).
Blinding	11a	If done, who was blinded after assignment to interventions (for example, participants, care providers, those assessing outcomes) and how	Included in primary publication which is referenced (reference 11).
	11b	If relevant, description of the similarity of interventions	Not applicable
Statistical methods	12a	Statistical methods used to compare groups for primary and secondary outcomes	Pages 7 and 8
	12b	Methods for additional analyses, such as subgroup analyses and adjusted analyses	Pages 7 and 8
**Results**			
Participant flow (a diagram is strongly recommended)	13a	For each group, the numbers of participants who were randomly assigned, received intended treatment, and were analysed for the primary outcome	Page 9, Table 1, Table S1 and Figure S1; also included in the primary publication which is referenced (reference 11)
	13b	For each group, losses and exclusions after randomisation, together with reasons	Figure S1; also included in the primary publication which is referenced (reference 11)
Recruitment	14a	Dates defining the periods of recruitment and follow-up	Page 4 ; also included in the primary publication which is referenced (reference 11)

	14b	Why the trial ended or was stopped	Included in primary publication which is referenced (reference 11).
Baseline data	15	A table showing baseline demographic and clinical characteristics for each group	Page 9, Table 1, Table S1 and Figure S1
Numbers analysed	16	For each group, number of participants (denominator) included in each analysis and whether the analysis was by original assigned groups	Page 9 and 10, tables 2, 3, S2, S3, S4 and S5, and Figures 1, 2 and S4
Outcomes and estimation	17a	For each primary and secondary outcome, results for each group, and the estimated effect size and its precision (such as 95% confidence interval)	Page 9 and 10, tables 2, 3, S2, S3, S4 and S5, and Figures 1, 2 and S4
	17b	For binary outcomes, presentation of both absolute and relative effect sizes is recommended	Page 9 and 10, tables 2, 3, S2, S3, S4 and S5, and Figures 1, 2 and S4
Ancillary analyses	18	Results of any other analyses performed, including subgroup analyses and adjusted analyses, distinguishing pre-specified from exploratory	Page 9 and 10, tables 3, S2, S3, S4 and S5, and Figures 1, 2 and S4
Harms	19	All important harms or unintended effects in each group (for specific guidance see CONSORT for harms)	Not applicable
**Discussion**			
Limitations	20	Trial limitations, addressing sources of potential bias, imprecision, and, if relevant, multiplicity of analyses	Page 13
Generalisability	21	Generalisability (external validity, applicability) of the trial findings	Pages 12 and 13
Interpretation	22	Interpretation consistent with results, balancing benefits and harms, and considering other relevant evidence	Pages 11-13
**Other information**			
Registration	23	Registration number and name of trial registry	Page 4
Protocol	24	Where the full trial protocol can be accessed, if available	Pages 4-8 ; also included in the primary publication which is referenced (reference 11)
Funding	25	Sources of funding and other support (such as supply of drugs), role of funders	Pages 14-17

Citation: Schulz KF, Altman DG, Moher D, for the CONSORT Group. CONSORT 2010 Statement: updated guidelines for reporting parallel group randomised trials. BMC Medicine. 2010;8:18. © 2010 Schulz et al. This is an Open Access article distributed under the terms of the Creative Commons Attribution License (http://creativecommons.org/licenses/by/2.0), which permits unrestricted use, distribution, and reproduction in any medium, provided the original work is properly cited.

*We strongly recommend reading this statement in conjunction with the CONSORT 2010 Explanation and Elaboration for important clarifications on all the items. If relevant, we also recommend reading CONSORT extensions for cluster randomised trials, non-inferiority and equivalence trials, non-pharmacological treatments, herbal interventions, and pragmatic trials. Additional extensions are forthcoming: for those and for up-to-date references relevant to this checklist, see www.consort-statement.org.
